# Complete genome sequence of *Bacillus xiamenensis* B0331, a polycaprolactone (PCL)-degrading bacterium isolated from dumpsite soil in Bay, Laguna, Philippines

**DOI:** 10.1128/mra.00364-26

**Published:** 2026-05-18

**Authors:** Jeffrey N. Nacasabog, Walter A. Laviña, Richard D. Tambalo, Andrew D. Montecillo

**Affiliations:** 1 Microbiology Division, Institute of Biological Sciences, College of Arts and Sciences, University of the Philippines Los Baños 54729, Los Baños, Laguna, Philippines; 2 National Institute of Molecular Biology and Biotechnology, University of the Philippines Los Baños, Los Baños, Laguna, Philippines; DOE Joint Genome Institute, Berkeley, California, USA

**Keywords:** plastic biodegradation, polycaprolactone, *Bacillus*, *Bacillus xiamenensis*, plastic-degrading bacteria

## Abstract

We report the complete genome sequence of a polycaprolactone-degrading *Bacillus xiamenensis* B0331, comprising a 3.78 Mb chromosome and three plasmids (94.67, 6.48, and 5.99 kb). The strain was isolated from dumpsite soil in Bay, Laguna, Philippines.

## ANNOUNCEMENT

Polycaprolactone (PCL) is a model substrate for screening polyester-degrading microorganisms (1). Here, we report the complete genome sequence of PCL-degrading *Bacillus xiamenensis* B0331 isolated from dumpsite soil. This report expands the known functional diversity of this species and highlights its potential in plastic biodegradation.

**Fig 1:**
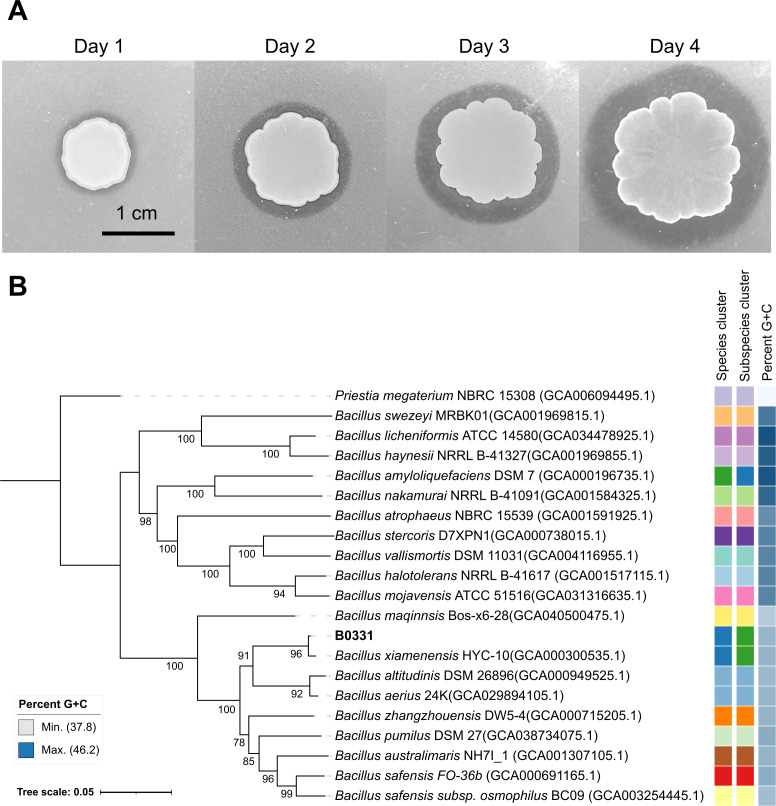
(**A**) Clear zone formation on PCA supplemented with 0.1% (w/v) PCL following spot inoculation of strain B0331, evident as early as day 1 and progressing through day 4. (**B**) Whole-genome phylogenomic tree showing the placement of strain B0331 among closely related *Bacillus* type strains. The tree was inferred using FastME 2.1.6.1 (5) from Genome BLAST Distance Phylogeny (GBDP) distances calculated via TYGS. Branch lengths are scaled according to GBDP distance formula d5, and values indicate pseudo-bootstrap support (100 replicates). The tree was rooted with *Priestia megaterium* NBRC 15308.

B0331 was isolated from dumpsite soil collected at Bay Central Materials Recovery Facility (MRF) in Laguna, Philippines (14°07′05″ N, 121°14′47″ E) in January 2025 (2). Selective enrichment was performed based on Yoshida et al. (3) with modifications: 1 mL of soil suspension (1 g soil in 9 mL PBS) was inoculated into 9 mL carbon-free basal medium with surface-sterilized 10 mm² PET plastic cut from a postconsumer bottle and incubated at 30°C for 10 days. The plastic and 1 mL culture were transferred to fresh medium and further incubated for 40 days. Isolates were screened on plate count agar (PCA) with 0.1% (wt/vol) PCL at 37°C (4). Isolate B0331 formed visible clear zones within 24 h of incubation (Fig. 1A).

Overnight cultures of B0331 grown in 10 mL Nutrient Broth (NB) at 37°C were harvested, washed with phosphate-buffered saline (PBS), and resuspended in 500 µL Zymo 1× DNA/RNA Shield and sent to Plasmidsaurus (Singapore) for hybrid genome sequencing. Genomic DNA was extracted with Zymo Quick-DNA Fungal/Bacteria Miniprep Kit (Zymo Research) according to the manufacturer’s instructions. The same genomic DNA extract was used for both long- and short-read library preparation. Long-read sequencing was performed on a PromethION P24 (R10.4.1 flow cell) using the Rapid Barcoding Kit 96 V14 (SQK-RBK114.96) and basecalled using Dorado v.1.4.0 in super-accurate mode. Short reads were generated on NovaSeq X Plus (2 × 150 bp) using the Illumina DNA Prep kit.

Sequencing produced 243,291 long reads (605.1 Mb; *N*_50_ of 4,379 bp) and 4,812,677 paired-end short reads (1.5 Gb). Long reads were filtered with Chopper v0.8.0 (6) (-q 10, -l 500), yielding 243,090 reads (604.8 Mb; *N*_50_ of 4,381 bp). Genome assembly was performed from the filtered long reads using Autocycler v.0.4.0 (7), integrating assemblies from Canu v2.3 (8), Flye v2.9.5 (9), Miniasm v0.3 (10), Necat v0.0.1 (11), NextDenovo v2.5.2 (12), and Raven v1.8.3 (13). Short reads were filtered using fastp (14). The consensus assembly was polished with long reads using Medaka v2.0.1 and short reads with Polypolish v0.6.1 (15) and Pypolca v0.4.0 (16, 17). Default parameters were used unless otherwise specified. Assembly statistics were generated using QUAST v5.3.0 (18), SeqKit v2.10.0 (19), and Samtools v1.13 (20). The assembly was reoriented using Dnaapler v1.3.0 (21). The final assembly consisted of four circular contigs totaling 3,888,035 bp with a GC content of 41.22% (Table 1). The assembly had an *N*_50_ of 3,780,882 bp. BUSCO analysis (22) indicated 99.8% complete and 0.2% fragmented BUSCOs. PGAP v6.10 (23) predicted 3,918 protein-coding genes, 81 tRNAs, 24 rRNA genes, and identified 4 CRISPR arrays. B0331 was identified as *B. xiamenensis*, with the closest genome being *B. xiamenensis* HYC-10 (GCF_000300535.1), sharing 99.15% average nucleotide identity (ANI) and 83.15% genome fraction using MiGA (24). Phylogenomic analysis using TYGS (25) confirmed its placement within the *B. xiamenensis* species cluster (Fig. 1B), supported by a dDDH value of 94.6% (d4), exceeding the 70% species threshold (26) and 79% subspecies threshold (27).

**TABLE 1:** General features of contigs in the *B. xiamenensis* B0331 genome assembly

Contig	Length (bp)	Molecule type^*a*^	G + C content (%)	Coverage (×)		No. of predicted protein-coding genes	No. of predicted hypothetical proteins
				Long reads	Short reads		
1	3,780,882	Chromosome	41.5	143	655	3,795	417
2	94,670	Plasmid	36	187	1,033	110	71
3	6,488	Plasmid	38.5	3,521	5,752	8	3
4	5,995	Plasmid	37.5	3,144	4,918	6	3

^*a*^ Molecule type was determined using MOB-recon (28).

## Data Availability

The complete genome sequence of *Bacillus xiamenensis* B0331 has been deposited in GenBank under accession number GCA_055597355.1. The BioProject accession number is PRJNA1426980, and the BioSample accession number is SAMN55900254. The raw sequencing data for both short reads and long reads used in the assembly have been deposited in the NCBI Sequence Read Archive (SRA) under accession numbers SRR37574138 and SRR37574139, respectively.
